# Identification and Verification of Molecular Subtypes with Enhanced Immune Infiltration Based on m6A Regulators in Cutaneous Melanoma

**DOI:** 10.1155/2021/2769689

**Published:** 2021-01-02

**Authors:** Yitong Lin, Shu Wang, Shirui Liu, Sha Lv, Huayang Wang, Fuqiu Li

**Affiliations:** ^1^Department of Dermatology, The Second Hospital of Jilin University, Changchun, Jilin Province, 130041, China; ^2^Department of Radiotherapy, The Second Hospital of Jilin University, Changchun, Jilin Province, 130041, China; ^3^Department of Dermatology, Xiang'an Hospital of Xiamen University, Xiamen, Fujian Province, 361101, China

## Abstract

**Background:**

As the most aggressive type of skin cancer, cutaneous melanoma (CM) is experiencing a rapidly rising mortality in recent years. Exploring potential prognostic biomarkers or mechanisms of disease progression therefore has a great significance for CM. The purpose of this study was to identify genetic markers and prognostic performance of N6-methyladenosine (m6A) regulators in CM.

**Method:**

Gene expression profiles, copy number variation (CNV), and single nucleotide polymorphism (SNP) data of patients were obtained from The Cancer Genome Atlas (TCGA) database.

**Results:**

Genomic variation and association analysis of gene expressions revealed a high degree of genomic variation in the presence of m6A-regulated genes. m6A patients with high-frequency genomic variants in the regulatory gene tended to develop a worse prognosis (*p* < 0.01). Unsupervised cluster analysis of the expression profiles of m6A-regulated genes identified three clinically distinct molecular subtypes, including degradation-enhanced subgroup and immune-enhanced subgroup, with significant prognostic differences (*p* = 0.046). A novel prognostic signature, which was established according to m6A-related characteristic genes identified through genome-wide expression spectrum, could effectively identify samples with poor prognosis and enhanced immune infiltration, and the effectiveness was also verified in the dataset of the chip.

**Conclusion:**

We identified genetic changes in the m6A regulatory gene in CM and related survival outcomes. The findings of this study provide new insights into the epigenetic understanding of m6A in CM.

## 1. Introduction

As the most aggressive type of skin cancer, cutaneous melanoma (CM) resulted in 287,723 new cases and 60,712 deaths worldwide in 2018 [1]. Although it accounts for only 4% of all skin cancer cases, it is far more dangerous than other skin cancers. The treatment and control of CM still remain less effective, though great efforts have been devoted to the improvements in managing advanced regional and metastatic CM [2]. UV exposure is known to be closely associated with the development of melanoma [3]. The incidence of CM shows regional variations such as ethnic skin phenotypes and differences in sun exposure [4]. A study demonstrated that the mortality of melanoma patients is rapidly increasing in recent years [5] and also pointed out the management of melanoma patients remains a complex and evolving subject [6]. Thus, exploring potential prognostic biomarkers or the mechanism of disease development is highly necessary for CM.

RNA methylation has become a common phenomenon and a key regulator of transcript expressions [7]. N6-Methyladenosine (m6A), which is methylated at the N6 position of adenosine, is considered to be the most common conserved internal transcriptional modifications of messenger RNAs (mRNAs), microRNA (miRNAs), and long noncoding RNAs (lncRNAs) [8]. The deposition of m6A is encoded by a methyltransferase complex involving three homologous factors, including methyltransferases (termed as “writers”), demethylases (termed as “erasers”), and recognition from m6A binding proteins (termed as “readers”). The m6A dysregulation consists of multiple cellular processes even in human cancers. It has been demonstrated that m6A mRNA demethylase FTO could regulate melanoma tumorigenicity and react to anti-PD-1 blockade [9]. The m6A methyltransferase METTL3 promotes osteosarcoma progression by regulating the m6A level of LEF1 [10]. Furthermore, METTL3 (an oncogene) maintains SOX2 expression via the m6A-IGF2BP2-dependent mechanism in colorectal carcinoma cells and therefore could serve as a potential biomarker for cancer prognosis prediction [11].

So far, however, little is known about the relationship between m6A-related genes and CM. The Cancer Genome Atlas (TCGA) database allows an easy access to human cancer data of gene expressions, copy number variation (CNVs), and single nucleotide polymorphism (SNPs), which all play important roles in the development and progression of human cancers [12]. However, the CNV and SNP of m6A-related genes remain unknown to us.

This retrospective study developed a novel gene signature based on m6A regulators in CM with data acquired from TCGA database and also analyzed the performance of the signature. By analyzing genomic variations and gene expression associations, we found that these m6A regulatory genes showed high genomic variations; interestingly, patients with m6A regulatory genes of high-frequency genomic variation tended to develop a worse prognosis (*p* < 0.01). Three clinical subtypes with different molecular characteristics and significant prognostic differences were identified by performing unsupervised clustering analysis on the expression profiles of m6A regulatory genes. m6A-related characteristic genes were determined based on genome-wide expression profiles. A new prognostic signature was then built for identifying subgroups with poor prognosis and enhanced immune infiltration, and its performance was validated in the training set and validation set. In summary, we determined genetic changes in the m6A regulatory genes in CM and patient survival outcomes. The findings of this study provide new insights into the epigenetic understanding of m6A in CM.

## 2. Materials and Methods

### 2.1. Data Resource and Processing

All CM clinical data, copy number variation (CNV), single nucleotide polymorphism (SNP), and mRNA expression data were retrieved from TCGA-Assembler of the TCGA website [13] and downloaded in May 2019. For transcriptome data, we obtained 472 samples and downloaded data of read counts. Data was normalized by R package *DESeq*. For SNP data, we obtained a total of 469 samples, and the downloaded data was processed by MuTect [14]. For CNV data, there were 940 samples acquired by R package *RTCGA*. Here, the “deletion” was defined as segment_mean < −0.2, and the “amplification” was defined as the segment_mean > 0.2. For clinical information data, initially, there were a total of 389 clinical samples; after integrating the data and excluding samples with a survival time shorter than 90 days, a total of 250 CM samples were finally decided for further analysis.

**Figure 1:**
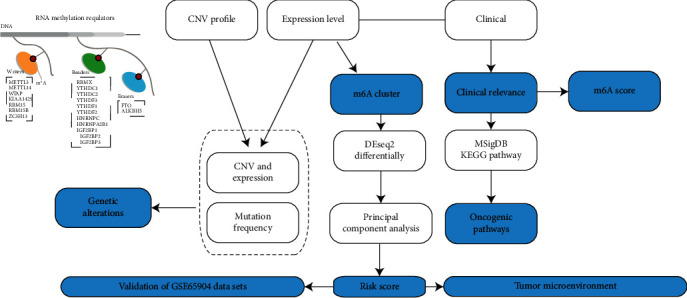
The work flow chart.

The expression spectrum dataset GSE65904 [15] with 214 melanoma samples from Illumina HumanHT-12 V4.0 Expression BeadChip platform was downloaded from the Gene Expression Omnibus (GEO) database. The samples with a survival time shorter than 90 days were selected, resulting in a final of 189 samples. Multiple probes corresponding to a gene were retained and shown as the median of the gene expression level, while probes corresponding to multiple genes were eliminated. Finally, the expression profile of genes was obtained. The work flow chart is shown in Figure 1.

### 2.2. Univariate Cox Survival Analysis

Here, the prognostic performance of m6A regulatory genes was examined. Based on the expression profile of m6A regulatory genes and clinical follow-up data of the samples, each m6A regulatory gene was evaluated using R software package survival, and the forest map was plotted applying R software package forestplot.

### 2.3. Clustering Analysis

For a better classification of the patients, an unsupervised clustering method was employed in hierarchical clustering analysis of the expression profile of CNV-related m6A regulatory genes. The clinical subtypes were selected according to the minimum intragroup variance and the maximum intergroup variance of the method.

### 2.4. Differential Expression Gene Analysis

Gene differences between each clinical subgroup were analyzed using R software package DESeq2, with ∣log2(fold change) | >2 and FDR < 0.05 serving as the thresholds.

### 2.5. Analysis of Immune Cells and Immune Infiltration

The ssGSEA (single-sample gene set enrichment analysis) algorithm was applied to quantify the relative abundance of cell invasion in each tumor microenvironment (TME) of a CM patient. The gene sets labeled as TME-infiltrating immune cell type were obtained from Charoentong et al.'s study [16], which investigated a variety of human immune cell subtypes, including activated CD8 T cells, activated dendritic cells, macrophages, natural killer T cells, and regulatory T cells. The enrichment fraction calculated by ssGSEA was used to represent the relative abundance of TME-infiltrating cells in the samples. Patient's infiltration score was assessed by the R package Estimate.

### 2.6. KEGG Pathway Enrichment Analysis of Different Clinical Subgroups

ssGSEA KEGG pathway analysis was performed on each sample using R software package GSVA [17] to calculate the difference of enrichment score of each pathway in different clinical subgroups. Moreover, the relationship between enrichment scores of each sample pathway and clinical subgroup was analyzed by performing Pearson correlation. *p* < 0.05 was the threshold value in determining the KEGG pathway that was the most relevant to the clinical subgroup.

### 2.7. Principal Component Analysis

Principal component analysis was performed according to the expression profile of m6A-related gene markers, with the first principal component being considered the scoring coefficient. A scoring model was established to calculate the risk score of each sample according to the model. Samples scored above 75% were assigned into the high-risk group with immunorejection phenotypes, while those scored below 75% were in the low-risk group.

### 2.8. Statistical Analysis

All statistical data were analyzed by SPSS 23 (IBM, Chicago, USA) and R language. The association between CNV and SNP of m6A regulatory genes and clinicopathological characteristics was examined by the chi-squared test. The association between three CM key genes and CNV and SNP of m6A regulatory genes was analyzed by the chi-squared test. The Kaplan-Meier curve and log-rank test were applied to evaluate the prognostic performance of the alterations in m6A regulatory genes. All statistical results with a *p* value ≤ 0.05 were considered to be significant.

## 3. Results

### 3.1. The m6A Regulatory Genes Showed a High Frequency of Genomic Variation in CM Patients

**Figure 2:**
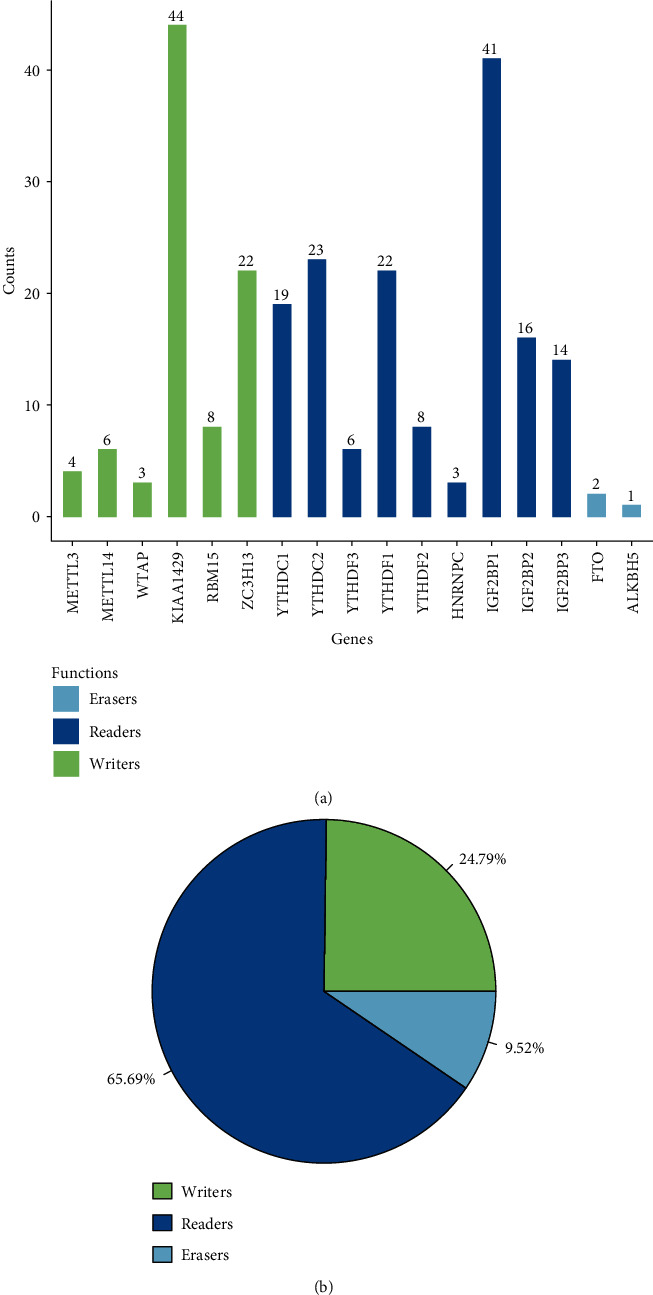
SNP and CNVs of m6A regulatory genes in CM patients: (a) frequency of SNP of different m6A regulatory genes in CM samples; (b) the CNV data of m6A regulatory genes in CM samples.

A total of 17 m6A regulatory genes were recruited in this study. 136 out of the 242 SNP of m6A regulatory genes with sequencing data appeared as independent samples (Table 1). Among them, the “writer” gene KIAA1429 in 44 samples and the “reader” gene IGF2BP1 in 41 samples demonstrated a higher SNP, with all the “reader” genes showing a higher SNP frequency than that of the “writer” or “eraser” genes; noticeably, the frequency of SNP of the “eraser” gene was the lowest (Figure 2(a)). We also observed that in all 469 CM samples with CNV data, the m6A regulatory genes had a high frequency of CNV (Figure 2(b)). For example, the “writer” gene WTAP showed the highest frequency of 57.08% of its CNV events, and the frequency of the “reader” gene IGF2BP3 was 52.1% (Table 2). These results indicated a high frequency of genomic SNP in the m6A regulatory gene in CM, and these abnormalities may affect gene expression and lead to different clinical outcomes in CM patients.

### 3.2. CNV of m6A Regulatory Genes Was Associated with Adverse Clinical Outcomes

**Figure 3:**
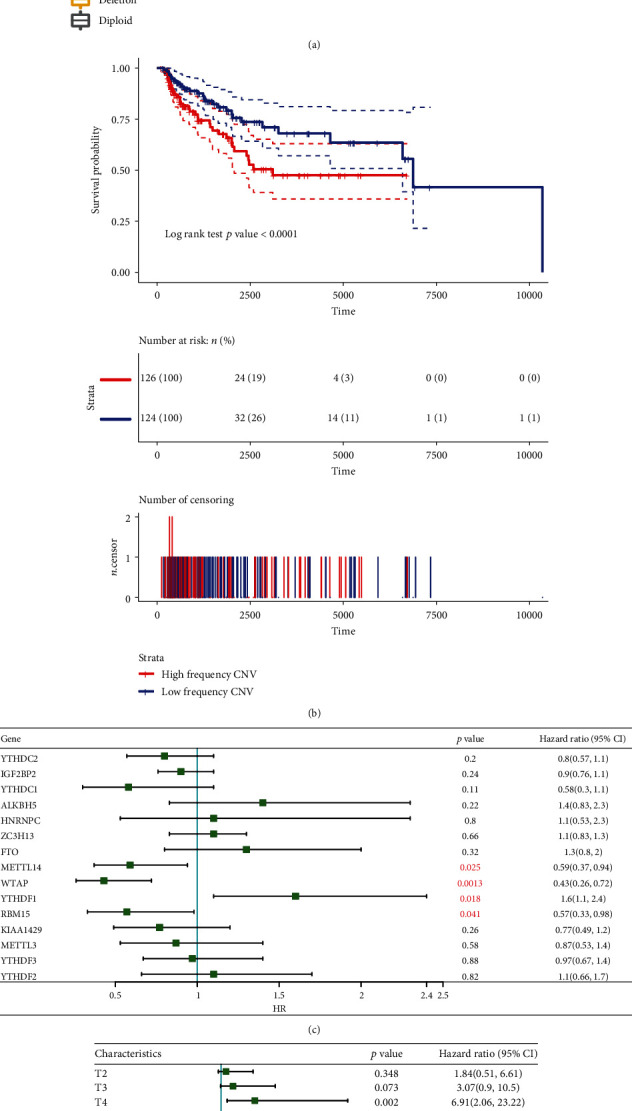
Relationship between m6A regulatory genes and CNV: (a) the relationships between CNV and expression levels of m6A regulatory genes. Kruskal test was used to detect the differences between different groups; (b) the Kaplan-Meier curves of CNV and prognosis in SCKM patients; (c) relationship between m6A regulatory genes and prognosis; (d) survival analysis by univariate Cox analysis on m6Ascore and clinical features; (e) survival analysis by multivariate survival Cox on m6Ascore and clinical features.

Considering that changes in CNV can affect gene expression levels through dosage compensation effects, to this end, the effect of m6A-regulated gene changes on mRNA expression was evaluated. In 472 KM samples, mRNA expression levels showed a significant correlation with CNV patterns. For all 17 regulatory genes, except for IGF2BP3 and IGF2BP1, increased CNV of the rest 15 genes was related to a higher mRNA expression and deletion in mRNAs with decreased expression (Figure 3(a)). Furthermore, the prognostic differences between samples of m6A regulatory genes with the high-frequency and low-frequency CNV abnormalities were analyzed. We observed that patients with a high CNV tended to show worse clinical outcomes (*p* < 0.001), directly indicating that abnormal copy number of m6A regulatory genes was related to poor clinical outcomes (Figure 3(b)). Finally, by analyzing the relationship between the expressions of m6A regulatory genes and patients' prognosis, we observed that RBM15, YTHDF1, WTAP, and METTL14 genes were significantly associated with CM prognosis (Figure 3(c)). Specifically, high-expressed YTHDF1 was indicative of a poor prognosis and could therefore be considered a risk factor, at the same time, low-expressed RBM15, WTAP, and METTL14 were also related to a poor prognosis and were all seen as protective factors. In order to further determine the prognostic value of m6A regulators, a risk score model (m6AScore) was established based on a multivariate regression method to calculate the risk score of each patient in TCGA cohort, and the prognostic value of m6AScore was determined by univariate and multivariate Cox survival analyses. The results of univariate analysis demonstrated that m6AScore and a variety of clinical features such as T, N, and stages were strongly correlated with CM patients' prognosis (Figure 3(d)), while multivariate analysis showed that only m6AScore, N3, N2, and T4 had a significant prognostic correlation (Figure 3(e)). In addition, HR of N3, N2, and T4 was abnormally high. These results indicated that m6AScore has a more stable prognostic performance than T, N, M, and stage or other clinical features.

### 3.3. Different Molecular Subgroups Identified by Unsupervised Cluster Analysis Based on Expressions of m6A Genes

**Figure 4:**
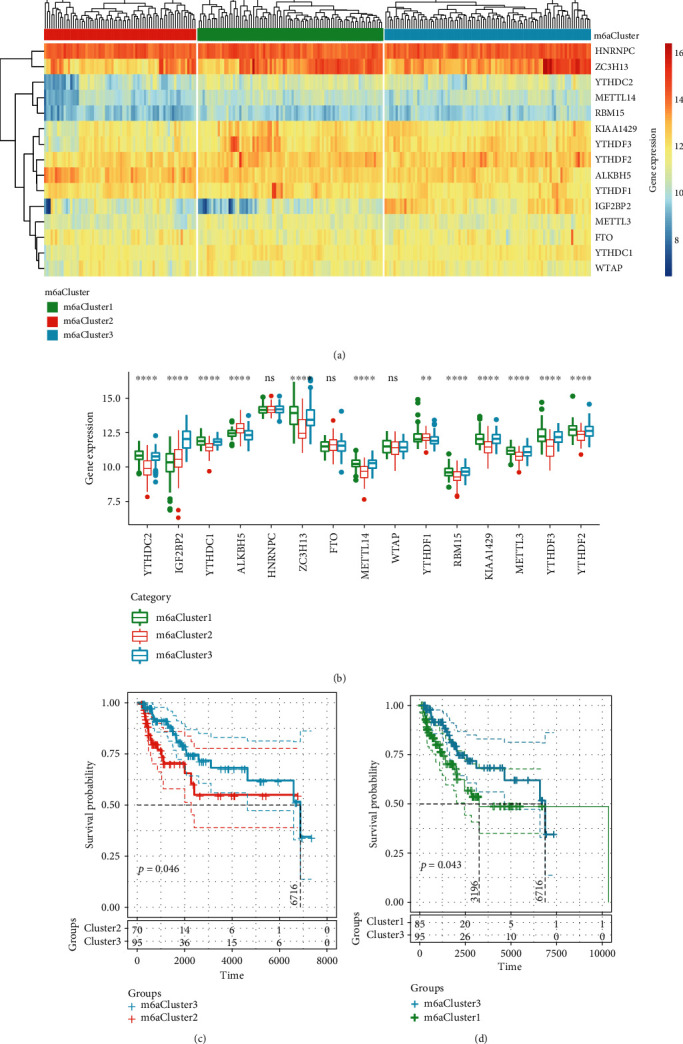
Different molecular subgroups identified by unsupervised cluster analysis based on m6A gene expression: (a) identification of molecular subtypes; (b) heat map of differentially expressed genes; (c) KM prognosis curve of m6ACluster2 and m6ACluster3; (d) KM prognosis curve of m6ACluster1 and m6ACluster3.

Given that m6A regulatory genes are associated with CM prognosis, unsupervised hierarchical cluster analysis was conducted based on the expression profiles of 15 CNV-related m6A regulatory genes. We observed that these 15 genes were divided into three main categories (Figure 4(a)) with different expression patterns of m6A genes. Further analysis showed that 12 genes (80%) out of the 15 genes in the three subtypes had significant expression differences (Figure 4(b)). Subsequently, prognostic differences among the three types of samples were examined, and the data demonstrated that m6A Cluster3 was related to a significantly better prognosis than m6A Cluster1 and m6A Cluster2 (Figures 4(c) and 4(d)). These results suggest that the three m6A Clusters have different molecular characteristics, which will lead to different clinical outcomes.

### 3.4. The Regulatory Characteristics of m6A Cluster

**Figure 5:**
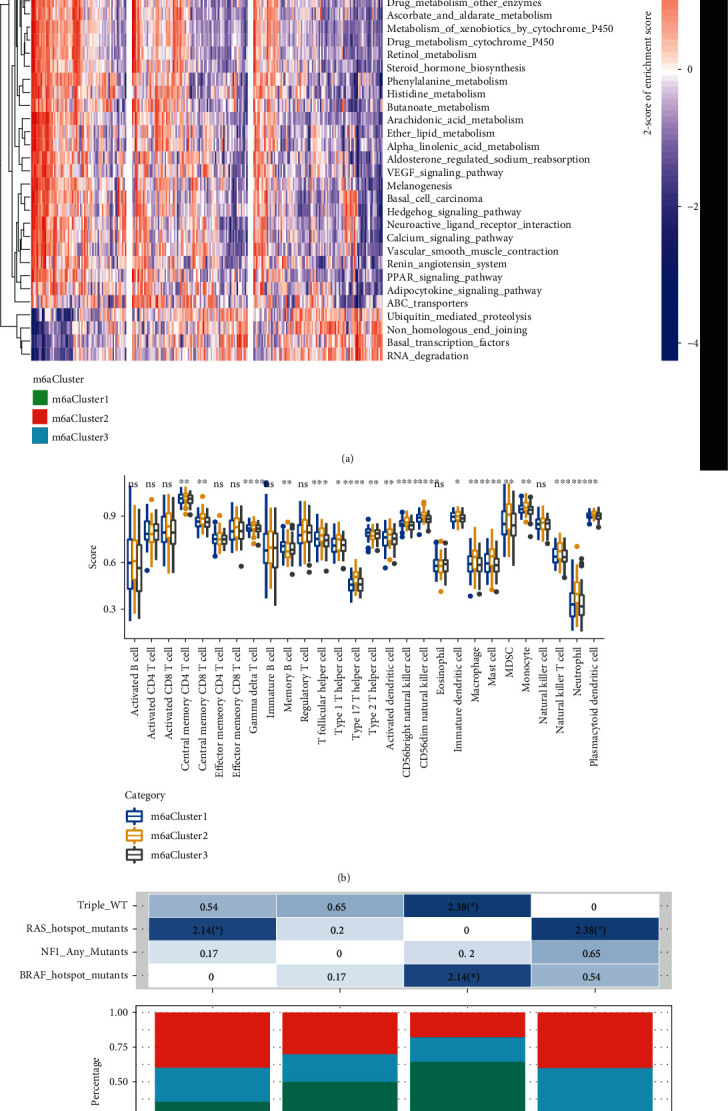
The regulatory characteristics of m6ACluster: (a) enrichment fraction heat map of the KEGG pathway in each sample with significant enrichment; (b) the score distribution of 28 immune cells in different m6AClusters; (c) the intersection relationship between the three m6AClusters and the four genomic isoforms.

To explore the relationship between the biological behaviors of CM and m6A Cluster, the enrichment scores of each sample in the KEGG pathway were calculated by ssGSEA to further determine the differences of the enrichment scores (Figure 5(a)). Significant differences in 44 KEGG pathways can be observed; interestingly, 40 pathways showed significant positive correlations with m6A Cluster2, and they were mainly important signaling pathways involved in tumor development, for example, VEGF_signaling_pathway, basal_cell_carcinoma, and drug_metabolism_cytochrome. In addition, the remaining 4 KEGG pathways, namely, ubiquitin_mediated_proteolysis, non_homologous_end_joining, basal_transcription_factors, and RNA_degradation, were significantly upregulated in m6A Cluster3 and are mainly related to the degradation process. 28 immune cell components were calculated for each patient by the ssGSEA, and a significant difference in the distribution of 19 immune cells (67.9%) was detected (Figure 5(b)). Among these significant immune cells, memory B cell and type 2 T helper cell showed the highest scores in m6A Cluster1, while the remaining 17 immune cells all had the highest scores in m6A Cluster2. These results suggested that m6A Cluster3 is related to immune processes and a better CM prognosis, while m6A Cluster2 is related to a stable immune microenvironment and a worse prognosis. We also compared the relationships between the 3 m6A-related molecular subtypes and the previously reported genomic subtypes (BRAF, RAS, NF1, and triple-WT) [18]). The data showed that m6A Cluster3 had the largest intersection with the RAS subtype, whereas m6A Cluster1 and m6A Cluster2 had the largest intersection with triple_WT (Figure 5(c)). In our study, the results that m6A Cluster1 and m6A Cluster2 were indicative of a poorer prognosis and higher frequency of m6A copy number are consistent with triple-WT with significantly more copy number fragments.

### 3.5. Identification of m6A Gene Markers and Molecular Characteristics

**Figure 6:**
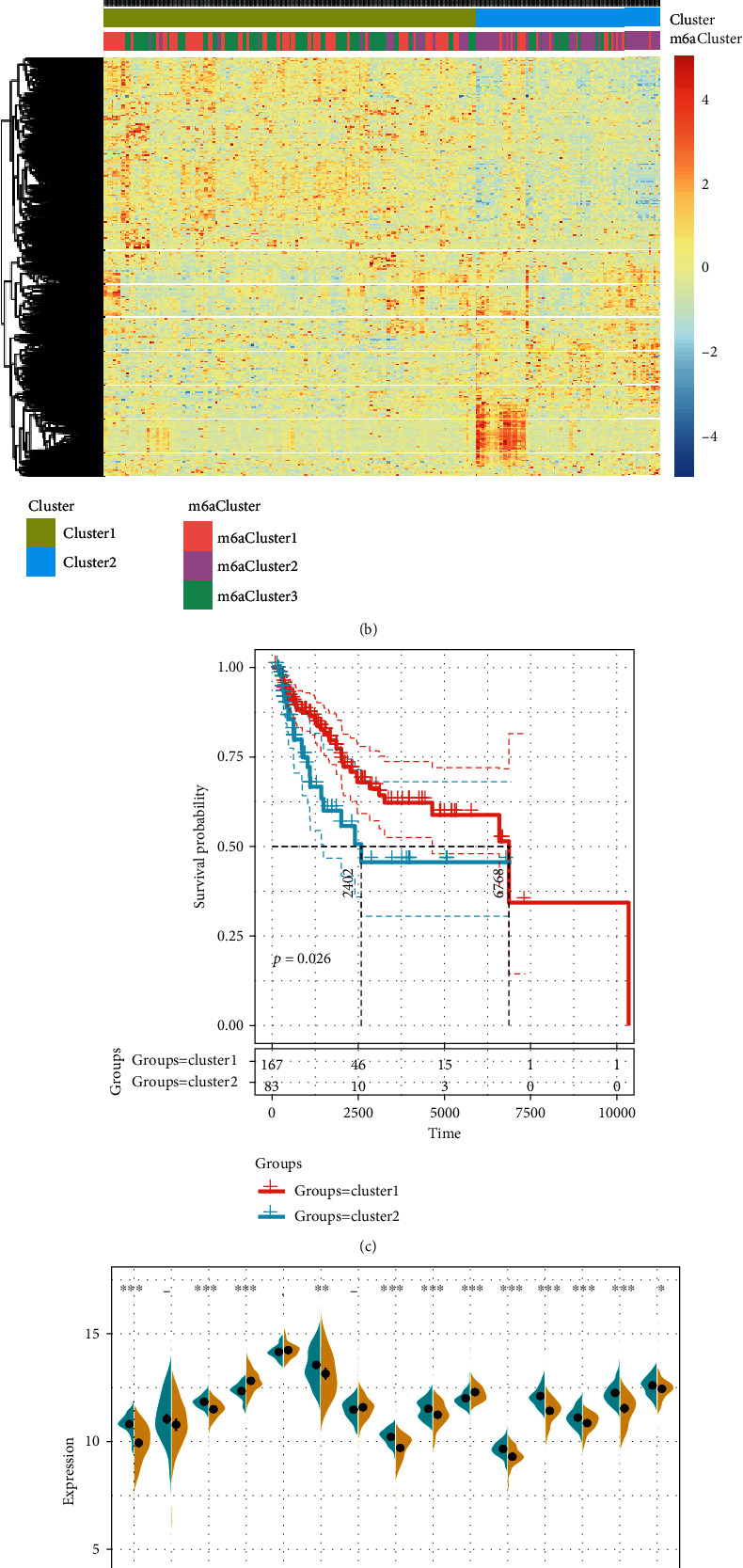
Identification of m6A gene markers and molecular characteristics: (a) Wayne diagram of differential genes in m6AClusters; (b) heat map of differentially expressed genes in m6AClusters; (c) prognostic KM curve of Cluster1 and Cluster2; (d) the expression differences of 15 CNV-related m6A regulatory genes in Cluster1 and Cluster2.

To determine the gene expression differences among the m6A cluster samples, we identified 5,533 differentially expressed genes based on the gene expression profile. It has been found that m6A Cluster2 and m6A Cluste1/m6A Cluster3 had the most differentially expressed genes, as compared with m6A Cluster1 (Figure 6(a)). Gene expression profiles of these 5533 genes were extracted for cluster analysis, and the data demonstrated that these genes divided the samples into two categories (Cluster1 and Cluster2). Specifically, Cluster2 mainly consisted of m6A Cluster2 samples and a small part of m6A Cluster3 samples, Cluster1 contained almost all the m6A Cluster1 samples, and Cluster1 contained most of the m6A Cluster3 samples (Figure 6(b)). Further analysis demonstrated that Cluster2 was related to a significantly worse prognosis than Cluster1 (Figure 6(c)). Moreover, we also detected the expression differences of 15 m6A regulatory genes with CNV abnormalities in Cluster1 and Cluster2 (Figure 6(d)), and 12 (80%) out of the 15 genes showed significant expression differences, and among the 12 genes, ALKBH5 and YTHDF1 were significantly highly expressed in Cluster2, while the remaining 10 genes were significantly highly expressed in Cluster1. These results indicated that a class of molecular subgroups (m6A Cluster1/Cluster2) with unfavorable prognosis which resulted from abnormalities of immune microenvironment could be identified by m6A regulatory genes or m6A-related gene markers.

### 3.6. Establishment and Verification of Prognostic Signature Based on m6A Regulatory Genes

**Figure 7:**
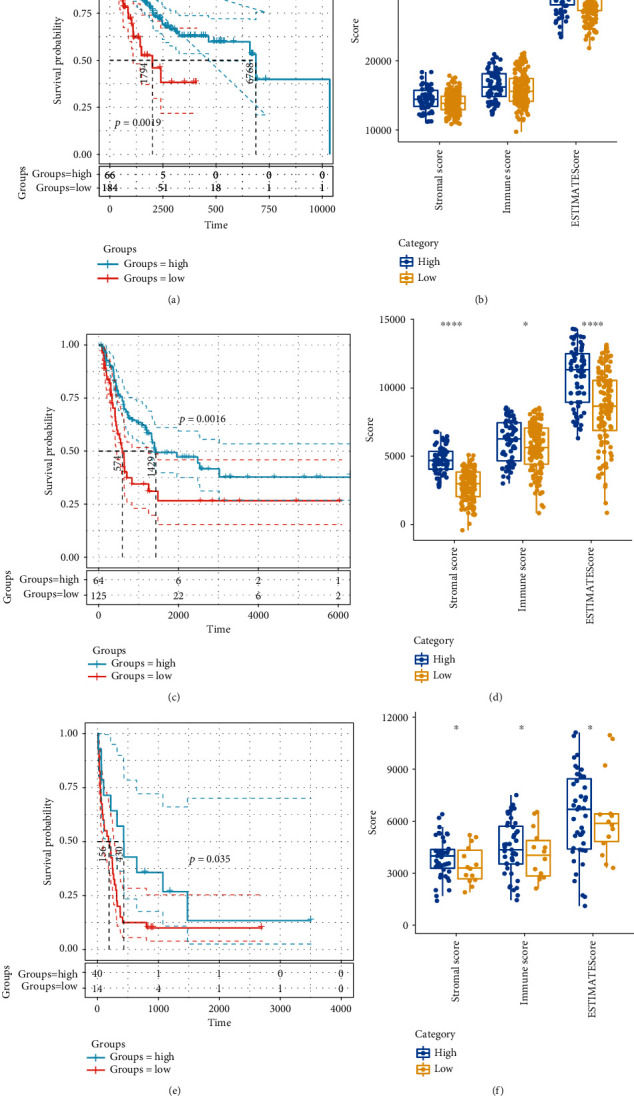
Establishment and validation of risk scores based on m6A-related genetic markers: (a) KM curves for prognostic differences between high-risk and low-risk groups; (b) differences in immune microenvironment scores between high-risk and low-risk groups; (c) KM curves for prognostic differences between high-risk and low-risk groups in the GSE65904 dataset; (d) differences in immune microenvironment scores between high-risk and low-risk groups in the GSE65904 dataset; (e) KM curves for prognostic differences between high-risk and low-risk groups in the GSE22153 dataset; (f) differences in immune microenvironment scores between high-risk and low-risk groups in the GSE22153 dataset.

The subgroup of immune-related prognosis was determined by performing the principal component analysis based on the expression profiles of m6A-related gene markers to divide the samples into a high-risk group and low-risk group. A careful analysis of the prognostic differences between the two groups showed that the prognosis in the high-risk group was significantly worse than the low-risk group (Figure 7(a)). Moreover, a higher immune score was observed in the high-risk group, as compared with the low-risk group (Figure 7(b)). Furthermore, the patients were accordingly group based on the chip platform GSE65904. Here, the data showed that patients' prognosis in the high-risk group sample was greatly more unfavorable than that in the low-risk group (Figure 7(c)). Immunomicroenvironment analysis demonstrated that the samples from the high-risk group had higher immune microenvironment scores (Figure 7(d)). In addition, the Swegene Center for Integrative Biology at Lund University melanoma cohort (GSE22153) was introduced for verification. It has been found that the prognosis of the high-risk group was significantly worse than that of the low-risk group samples (Figure 7(e)). Immunomicroenvironment analysis showed that the high-risk group samples had a higher immune microenvironment score (Figure 7(f)). These results suggested that the expression profiles of m6A-related gene markers could serve as prognostic markers to evaluate the prognosis of CM patients.

## 4. Discussion

The present study developed a novel gene signature based on m6A regulators to CM using TCGA database and also assessed its predictive performance. Genetic alterations of the m6A regulatory genes have been found to be closely related to CM patients' survival outcomes. Our findings provide new insights into the epigenetic understanding of m6A in CM.

As the most common internal chemical modification of mRNA, m6A is widely involved in many pathological processes of cancer development. The deposition of m6A is encoded by a methyltransferase complex involving three homologous factors including methyltransferases (such as METTL3/14, WTAP, RBM15/15B, and KIAA1429, termed as “writers”), demethylases (such as FTO and ALKBH5, termed as “erasers”) and recognition from m6A binding proteins (such as YTHDF1/2/3, IGF2BP1 and HNRNPA2B1, termed as “readers”) [8]. Specifically, METTL3 silencing reduces m6A methylation and downregulates the total mRNA level of lymphoid enhancer binding factor 1 (LEF1), thereby inhibiting the activity of the Wnt/*β*-catenin signaling pathway. Moreover, METTL3 promotes osteosarcoma cell progression by regulating the m6A level of LEF1 and activating the Wnt/*β*-catenin signaling pathway [10]. Previously, upregulated expression of METTL3 has been observed in human hepatocellular carcinoma and multiple solid tumors [19]. Knockdown of METTL3 can reduce hepatocellular carcinoma cell proliferation, migration, and colony formation, and more interestingly, knockout of METTL3 remarkably suppresses tumorigenicity and lung metastasis. In colorectal cancer, METTL3 acts as a tumor suppressor on cell proliferation, migration, and invasion via the p38/ERK pathway [20]. In addition, reducing m6A methylation could activate oncogenic Wnt/PI3K-Akt signaling and promote malignant phenotypes of gastric cancer cells [21]; moreover, METTL3 knockdown could inhibit the level of total RNA methylation of m6A, cell proliferation, and migration of gastric cancer cells [22]. Noticeably, m6A methylation has also been reported in bladder cancer [23], renal cancer [24], acute myeloid leukemia [25], breast cancer [26], and hepatocellular cancer [27].

The regulators of m6A RNA methylation function critically in the malignant progression of glioma and may be useful for the development of prognostic stratification and therapeutic strategies [28]. In this study, 15 m6A regulatory genes with abnormal copies of the genome were identified based on TCGA gene expression data and CNV data. Our analysis showed that a higher frequency of the CNV of m6A regulatory genes was related to a worse the prognosis of CM patients. Three clinical subgroups with different molecular characteristics were detected after performing unsupervised cluster analysis. Here, m6aCluster2 was associated with abnormal immune microenvironment and poor prognosis. A risk assessment model was established with the m6A-related gene markers, and subgroups with high immune scores and poor prognosis were identified in both sequencing data and chip data. Our results indicated that m6A regulatory genes play an important role in CM, as they could influence immune-related processes during tumor development. Zhou et al. [29] proved that a low expression of the writer gene METTL3 is related to activations of adipogenesis and mTOR pathways. However, m6A regulators in CM have not been reported before.

Some cancer-related pathways are dysregulated during CM development. In this study, We determined a molecular subgroup with favorable prognosis based on m6A regulatory genes and found that degradation pathways such as ubiquitin pathways, mediated_proteolysis, and RNA_degradation were active in the subgroups, and interestingly, these pathways play critical roles in regulating cell growth and proliferation by controlling the abundance of key cyclins [30]. Evidences increasingly showed that abnormal proteolysis of cell cycle regulators significantly promotes tumorigenesis, while enhanced degradation of cell cycle regulators (i.e., protooncoproteins) can inhibit tumor metastasis. The driving force of the cell cycle is the activation of cyclin-dependent kinases (CDKs), whose activity is controlled by the proteolysis of ubiquitin-mediated key regulators (such as cyclins and CDK inhibitors), and disorder of proteolytic system may lead to uncontrolled proliferation, genomic instability, and cancer initiation [31]. Selective RNA degradation has been shown to be able to strongly resist disease development and is therefore believed to have the potential to treat cancer [32]. In antioral tumor therapy, activation of peroxisomal proliferator activator (PPAR) upregulates RNA degradation pathways, leading to reprogramming of tumor metabolism [33]. These results suggest that degradation-enhancing molecular subgroup identified by the m6A regulatory genes could be explored as a novel effective strategy for CM treatment.

## 5. Conclusion

In summary, this study first detected the genetic alterations of m6A regulatory genes in CM and identified molecular subsets with enhanced degradation and immune enhancement based on genomic mutagenesis of m6A regulatory genes. A m6A signature with a high predictive accuracy in predicting the prognosis of CM patients was constructed in this study. Our findings provide new insights into the epigenetic understanding of m6A in CM.

## Figures and Tables

**Table 1:** SNP of m6A regulatory genes.

Samples	Writers						Readers									Erasers	
	METTL3	METTL14	WTAP	KIAA1429	RBM15	ZC3H13	YTHDC1	YTHDC2	YTHDF3	YTHDF1	YTHDF2	HNRNPC	IGF2BP1	IGF2BP2	IGF2BP3	FTO	ALKBH5
TCGA-BF-A1Q0							p.P493L										
TCGA-BF-A3DJ															p.P574H		
TCGA-BF-A3DM								p.P1264S									
TCGA-BF-A5ES						p.T489T											
TCGA-BF-AAOX													p.P403S				
TCGA-BF-AAP1								p.P240S									
TCGA-BF-AAP4						p.P365S						—					
TCGA-BF-AAP7						p.R874C											
TCGA-D3-A1Q4				p.S1444R													

**Table 2:** Different CNV patterns occur in CM patients.

Types	Genes	Diploid	Deletion	Amplification	CNV sum	Amplification (%)	Deletion (%)	Percentage
Writers	METTL3	338	93	40	133	30.08%	69.92%	28.24%
	METTL14	354	76	41	117	35.04%	64.96%	24.84%
	WTAP	203	246	24	270	8.89%	91.11%	57.08%
	KIAA1429	268	10	194	204	95.10%	4.90%	43.22%
	RBM15	236	70	75	145	51.72%	48.28%	38.06%
	ZC3H13	303	76	92	168	54.76%	45.24%	35.67%
Readers	YTHDC1	361	68	52	120	43.33%	56.67%	24.95%
	YTHDC2	324	100	47	147	31.97%	68.03%	31.21%
	YTHDF3	282	14	175	189	92.59%	7.41%	40.13%
	YTHDF1	271	3	198	201	98.51%	1.49%	42.58%
	YTHDF2	346	55	70	125	56.00%	44.00%	26.54%
	HNRNPC	338	93	40	133	30.08%	69.92%	28.24%
	IGF2BP1	3342	43	90	133	67.67%	32.33%	3.83%
	IGF2BP2	358	51	62	113	54.87%	45.13%	23.99%
	IGF2BP3	228	18	230	248	92.74%	7.26%	52.10%
Erasers	FTO	329	116	36	152	23.68%	76.32%	31.60%
	ALKBH5	336	209	26	235	11.06%	88.94%	41.16%
Total		8217	1341	1492	2833	52.67%	47.33%	25.64%

## Data Availability

The datasets analyzed in this study are available from the GEO repository (https://www.ncbi.nlm.nih.gov/geo/).

## References

[1] Bray F., Ferlay J., Soerjomataram I., Siegel R. L., Torre L. A., Jemal A. (2018). Global cancer statistics 2018: GLOBOCAN estimates of incidence and mortality worldwide for 36 cancers in 185 countries. *CA: A Cancer Journal for Clinicians*.

[2] Gorayski P., Burmeister B., Foote M. (2015). Radiotherapy for cutaneous melanoma: current and future applications. *Future Oncology*.

[3] Sample A., He Y. Y. (2018). Mechanisms and prevention of UV-induced melanoma. *Photodermatology, Photoimmunology & Photomedicine*.

[4] Leonardi G. C., Falzone L., Salemi R. (2018). Cutaneous melanoma: from pathogenesis to therapy (review). *International Journal of Oncology*.

[5] Eggermont A. M., Spatz A., Robert C. (2014). Cutaneous melanoma. *The Lancet*.

[6] Pavri S. N., Clune J., Ariyan S., Narayan D. (2016). Malignant melanoma: beyond the basics. *Plastic and Reconstructive Surgery*.

[7] Lan Q., Liu P. Y., Haase J., Bell J. L., Huttelmaier S., Liu T. (2019). The critical role of RNA m6A methylation in cancer. *Cancer Research*.

[8] Chen X. Y., Zhang J., Zhu J. S. (2019). The role of m6A RNA methylation in human cancer. *Molecular Cancer*.

[9] Yang S., Wei J., Cui Y. H. (2019). m(6)A mRNA demethylase FTO regulates melanoma tumorigenicity and response to anti-PD-1 blockade. *Nature Communications*.

[10] Miao W., Chen J., Jia L., Ma J., Song D. (2019). The m6A methyltransferase METTL3 promotes osteosarcoma progression by regulating the m6A level of LEF1. *Biochemical and Biophysical Research Communications*.

[11] Li T., Hu P. S., Zuo Z. (2019). METTL3 facilitates tumor progression via an m6A-IGF2BP2-dependent mechanism in colorectal carcinoma. *Molecular Cancer*.

[12] Gigek C. O., Calcagno D. Q., Rasmussen L. T. (2017). Genetic variants in gastric cancer: risks and clinical implications. *Experimental and Molecular Pathology*.

[13] Weinstein J. N., The Cancer Genome Atlas Research Network, Collisson E. A. (2013). The Cancer Genome Atlas pan-cancer analysis project. *Nature Genetics*.

[14] Cibulskis K., Lawrence M. S., Carter S. L. (2013). Sensitive detection of somatic point mutations in impure and heterogeneous cancer samples. *Nature Biotechnology*.

[15] Cabrita R., Lauss M., Sanna A. (2020). Tertiary lymphoid structures improve immunotherapy and survival in melanoma. *Nature*.

[16] Charoentong P., Finotello F., Angelova M. (2017). Pan-cancer immunogenomic analyses reveal genotype-immunophenotype relationships and predictors of response to checkpoint blockade. *Cell Reports*.

[17] Hanzelmann S., Castelo R., Guinney J. (2013). GSVA: gene set variation analysis for microarray and RNA-seq data. *BMC Bioinformatics*.

[18] Akbani R., Akdemir K. C., Aksoy B. A. (2015). Genomic classification of cutaneous melanoma. *Cell*.

[19] Chen M., Wei L., Law C. T. (2018). RNA N6-methyladenosine methyltransferase-like 3 promotes liver cancer progression through YTHDF2-dependent posttranscriptional silencing of SOCS2. *Hepatology*.

[20] Deng R., Cheng Y., Ye S. (2019). m(6)A methyltransferase METTL3 suppresses colorectal cancer proliferation and migration through p38/ERK pathways. *Oncotargets and Therapy*.

[21] Zhang C., Zhang M., Ge S. (2019). Reduced m6A modification predicts malignant phenotypes and augmented Wnt/PI3K-Akt signaling in gastric cancer. *Cancer Medicine*.

[22] Liu T., Yang S., Sui J. (2019). Dysregulated N6-methyladenosine methylation writer METTL3 contributes to the proliferation and migration of gastric cancer. *Journal of Cellular Physiology*.

[23] Han J., Wang J. Z., Yang X. (2019). METTL3 promote tumor proliferation of bladder cancer by accelerating pri-miR221/222 maturation in m6A-dependent manner. *Molecular Cancer*.

[24] Gong D., Zhang J., Chen Y. (2019). The m6A-suppressed P2RX6 activation promotes renal cancer cells migration and invasion through ATP-induced Ca2+ influx modulating ERK1/2 phosphorylation and MMP9 signaling pathway. *Journal of Experimental & Clinical Cancer Research*.

[25] Ianniello Z., Paiardini A., Fatica A. (2019). N6-Methyladenosine (m6A): a promising new molecular target in acute myeloid leukemia. *Frontiers in Oncology*.

[26] Wu L., Wu D., Ning J., Liu W., Zhang D. (2019). Changes of N6-methyladenosine modulators promote breast cancer progression. *BMC Cancer*.

[27] Cheng X., Li M., Rao X. (2019). KIAA1429 regulates the migration and invasion of hepatocellular carcinoma by altering m6A modification of ID2 mRNA. *Oncotargets and Therapy*.

[28] Chai R. C., Wu F., Wang Q. X. (2019). m6A RNA methylation regulators contribute to malignant progression and have clinical prognostic impact in gliomas. *Aging (Albany NY)*.

[29] Zhou J., Wang J., Hong B. (2019). Gene signatures and prognostic values of m6A regulators in clear cell renal cell carcinoma - a retrospective study using TCGA database. *Aging (Albany NY)*.

[30] Bashir T., Pagano M. (2003). Aberrant ubiquitin-mediated proteolysis of cell cycle regulatory proteins and oncogenesis. *Advances in Cancer Research*.

[31] Nakayama K. I., Nakayama K. (2006). Ubiquitin ligases: cell-cycle control and cancer. *Nature Reviews Cancer*.

[32] Dey S. K., Jaffrey S. R. (2019). RIBOTACs: small molecules target RNA for degradation. *Cell Chemical Biology*.

[33] Chang N. W., Huang Y. P. (2019). The RNA degradation pathway is involved in PPAR*α*-modulated anti-oral tumorigenesis. *Biomedicine*.

